# Targeted DNA sequencing and *in situ* mutation analysis using mobile phone microscopy

**DOI:** 10.1038/ncomms13913

**Published:** 2017-01-17

**Authors:** Malte Kühnemund, Qingshan Wei, Evangelia Darai, Yingjie Wang, Iván Hernández-Neuta, Zhao Yang, Derek Tseng, Annika Ahlford, Lucy Mathot, Tobias Sjöblom, Aydogan Ozcan, Mats Nilsson

**Affiliations:** 1 Science for Life Laboratory, Department of Immunology, genetics and pathology, Uppsala University, Box 815, 751 08 Uppsala, Sweden; 2 Science for Life Laboratory, Department of Biophysics and biochemistry, Stockholm University, Sweden, Box 1031, Se-171 21 Solna, Sweden; 3 Electrical Engineering Department, University of California, Los Angeles, Los Angeles, California 90095, USA; 4 Bioengineering Department, University of California, Los Angeles, Los Angeles, California 90095, USA; 5 California NanoSystems Institute (CNSI), University of California, Los Angeles, Los Angeles, California 90095, USA; 6 Devyser AB, Instrumentvägen 19, 126 53 Stockholm, Sweden

## Abstract

Molecular diagnostics is typically outsourced to well-equipped centralized laboratories, often far from the patient. We developed molecular assays and portable optical imaging designs that permit on-site diagnostics with a cost-effective mobile-phone-based multimodal microscope. We demonstrate that targeted next-generation DNA sequencing reactions and *in situ* point mutation detection assays in preserved tumour samples can be imaged and analysed using mobile phone microscopy, achieving a new milestone for tele-medicine technologies.

## Abstract (web summary)

On-site diagnostics technologies allow for rapid, cost-effective diagnosis with a particular importance for remote communities. Here the authors demonstrate the use of mobile phone based microscopy for targeted DNA sequencing and *in situ* point mutation detection in tumours.

Molecular diagnostics at the point of care (POC) is currently by and large an unmet need in resource-limited settings. Efficient management of a wide range of disease conditions is severely limited by the lack of molecular information. Here we focus our attention to the emerging field of molecular pathology, in which molecular analysis increasingly complements traditional morphology-based diagnosis of cancer for improved treatment precision^1^,^2^. Due to assay complexity and infrastructure requirements, such diagnostic assays are usually outsourced to specialized labs and sequencing centres. A parallel development is digital pathology, which enables pathologists to view samples and analyse molecular diagnostic data remotely, hence making the best use of the limited resource of professional pathology expertise. Mobile phones may further accelerate this emerging trend of tele-medicine and enable on-site implementation of digital pathology in resource-limited settings by supplying a cost-effective technology infrastructure for imaging and diagnostic analysis^3^. With their rapidly expanding imaging and sensing capabilities, computational power, and connectivity, mobile phones help translating biomedical measurements from lab environments to the POC and field settings^3^,^4^,^5^,^6^,^7^,^8^,^9^. For example, using the CMOS imager chips of mobile phone cameras, it is now possible to image tumour samples over large fields of view, with spatial resolution and image quality matching high-end pathology microscopes^3^.

Here we demonstrate a new milestone for mobile-phone-based biomolecular analysis and diagnostics that may facilitate transferring molecular diagnostic data from the POC to the point-of-expertise, providing a cost-effective means for molecular diagnostics even in resource-limited settings. We show that a cost-effective and compact multimodal microscope integrated on a mobile phone can be used for (i) targeted DNA sequencing and (ii) *in situ* point mutation analysis that allow integrating molecular analysis with tumour tissue morphology.

## Results

### Quantification of RCA products with mobile phone microscopy

**Figure 1:**
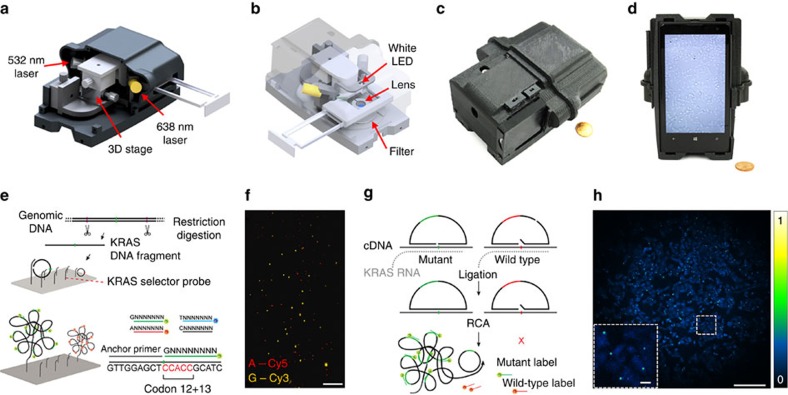
Multimodal mobile microscopy device and schematics of RCA assays. (**a**,**b**) 3D schematic illustration of the inner structure and the optical design of the mobile-phone-based microscopy platform. (**c**,**d**) Photographs of the mobile-phone-based microscope from different viewing perspectives. Mobile phone screen of (**d**) shows a bright-field image of fixated A549 cells captured by the phone. (**e**) DNA sequencing sample preparation scheme: genomic DNA is restriction digested and the *KRAS* DNA fragment selectively circularized on *KRAS* selector probes attached to slides. The DNA fragments are ligated and amplified on the slide, and the RCA products sequenced by unchained SBL chemistry^15^,^20^. DNA sequencing reactions are then imaged through our mobile phone microscope. (**f**) Dual-colour mobile phone microscope image of a targeted SBL reaction of *KRAS* codon 12 in genomic DNA extracted from A427 cells which are heterogeneous for a *KRAS* codon 12 mutation. RCPs are either stained with Cy3 corresponding to base G (*KRAS* wild type), or Cy5 corresponding to base A (*KRAS* mutant). Scale bar, 50 μm. (**g**) Schematic diagram of *in situ* point mutation detection assay through padlock probes and RCA. *KRAS* mRNA is converted to cDNA, which is targeted by single-base-discriminating padlock probes. Mutant specific padlock probes are ligated and amplified through RCA. Wild-type-specific probes do not ligate on mutated *KRAS* cDNA and generate no RCP. (**h**) A full field of view image of the A549 cell line with *in situ* RCA detected codon 12 point mutations, imaged with our mobile phone fluorescence microscope. Scale bar, 200 μm (full field of view); Scale bar, 20 μm (inset).

**Figure 2:**
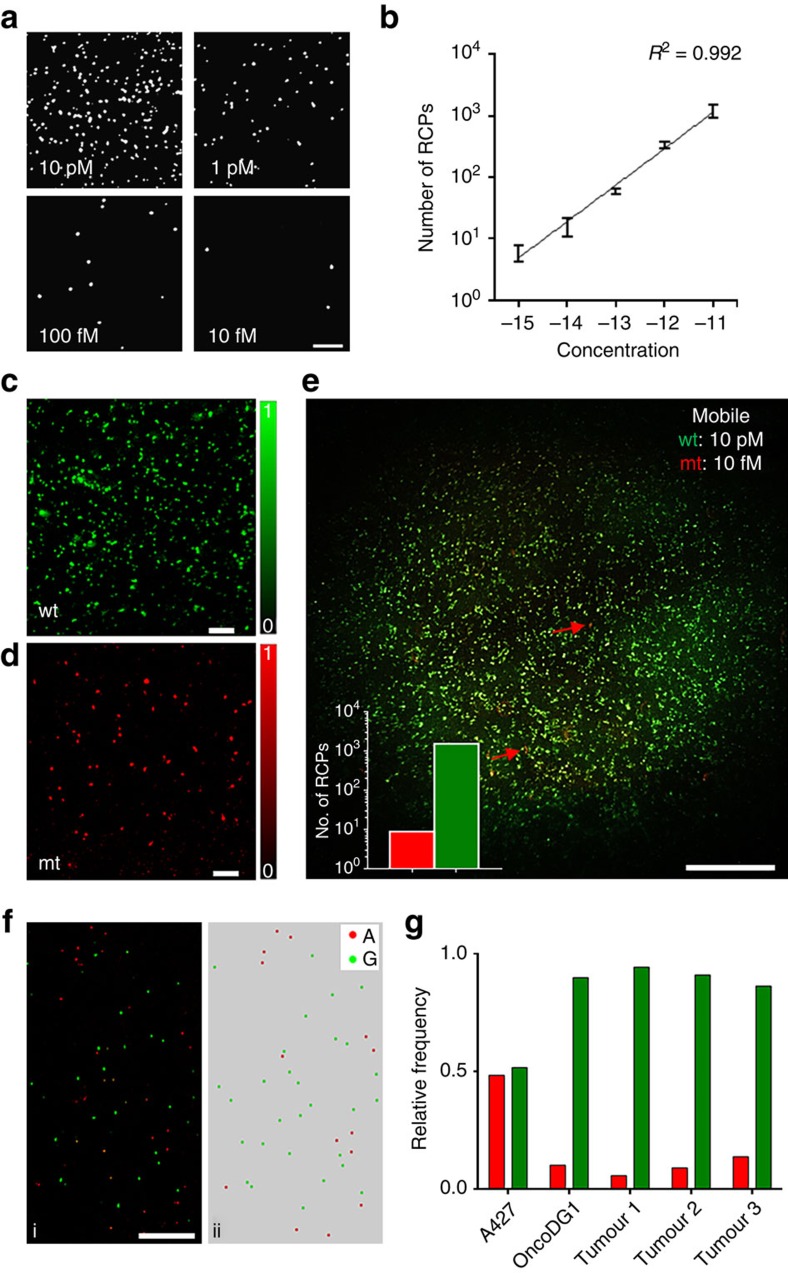
Mobile phone microscopy-based targeted DNA sequencing. (**a**) Amplified single-molecule detection through RCA and mobile phone microscopy: selected regions within images of 10 pM to 10 fM RCPs are depicted. Scale bar, 50 μm. (**b**) Quantification of RCPs generated from a log_10_ dilution series of synthetic circular templates. Error bars: 1 s.d. from the mean, *n*=3; linear regression is plotted as straight line. (**c**) *KRAS* wild type and (**d**) *KRAS* mutant (codon 12 mutation) synthetic DNA fragments were circularized through selector probes, amplified through RCA, and the first base in codon 12 sequenced by unchained SBL chemistry. Sequencing reactions were imaged at both of the fluorescent channels using the mobile phone microscope and these channels were digitally superimposed. Scale bar, 20 μm. (**e**) Synthetic *KRAS* fragments at a ratio of 1:1,000 mutant/wild type were sequenced and the reaction imaged through mobile phone microscopy. Cy3 stained RCPs (wild type—base G, green bar) and Cy5 stained RCPs (mutant—base A, red bar) were quantified and plotted in the inset graph. Red arrows in the mobile phone image point to RCPs that show a Cy5 sequencing signal. Scale bar, 200 μm. (**f**) Mobile phone microscopy-based sequence analysis: (i) fluorescence imaging of sequencing reaction, and (ii) base calling through a custom-written automated image analysis algorithm. Scale bar, 50 μm. (**g**) Quantification of sequencing experiments of extracted genomic DNA from cell lines and colon tumour biopsies using our mobile phone microscope. Relative frequencies of mutant (red bars) and wild type (green bars) RCPs are plotted.

First, we aimed to image individual rolling circle amplification (RCA)-amplified single molecules, generated on glass slides and inside preserved cells and tissues using a mobile-phone-based microscope. For these goals, we designed and 3D-printed a light-weight optomechanical attachment that is integrated with the existing camera module of a mobile phone (Fig. 1a–d). This optical attachment contains two compact laser diodes (at 532 and 638 nm) for multicolour fluorescence imaging (Fig. 1a) and a white light-emitting diode (LED) for bright-field transmission imaging (Fig. 1b,d). An integrated sample holder, consisting of a z-movement stage (Supplementary Fig. 1a, red part) and an x–y-movement stage (Supplementary Fig. 1a, blue part), enables three dimensional movement and alignment of the inserted sample slide. This optomechanical attachment to the mobile phone is also equipped with a cost-effective external lens module (focal length: 2.6 mm) providing a half-pitch resolution of 0.98 μm and an imaging field of view of ∼0.8 mm^2^ (Fig. 1; Supplementary Fig. 1b). For the molecular analysis, we developed targeted sequencing library preparation schemes based on selector probes (refs 10, 11) (Fig. 1e; Supplementary Note 1) and padlock probes *in situ* (refs 12, 13) (Fig. 1g), and RCA to generate micron-sized DNA coils that consist of hundreds of concatemerized repeats of the circular template and that can each be brightly labelled with fluorescent hybridization probes^14^ or sequenced^15^. We then established that individual RCA products (RCPs) can be discriminated and precisely quantified by our mobile-phone-based microscope over a 4-log dynamic range (1 fM–10 pM), demonstrating its utility to image and analyse individual RCA-amplified single molecules (Fig. 2a,b, Supplementary Software 1a).

### Targeted DNA sequencing for point mutation analysis

RCPs can be used as sequencing libraries in next-generation sequencing (NGS) applications^15^. To investigate whether our mobile-phone- based microscope can be used to read NGS reactions, we imaged and quantified sequencing by ligation (SBL) reactions of RCPs generated on standard microscopy slides (Fig. 2c–e, Supplementary Note 1, Supplementary Software 1b). To confirm that SBL reactions are base-specific, we generated sequencing libraries from synthetic *KRAS* fragments with either wild-type sequence, resulting in base G-specific Cy3 stain (Fig. 2c, Supplementary Fig. 2a), or with a codon 12 mutation, generating a base A-specific Cy5 stain (Fig. 2d, Supplementary Fig. 2b). To test the feasibility of detecting small amounts of mutant *KRAS* DNA within the background of wild-type DNA, synthetic fragments were sequenced in a ratio of 1:1,000 mutant:wild type (Fig. 2e). Our imaging results show that nine mutant RCPs with base A-specific stain (Cy5 labelled) were successfully detected using the mobile phone microscope among 1,552 wild-type RCPs with G-specific sequencing signal (Cy3 labelled) (Fig. 2e, inset). At such high sequencing depths (>1,000-fold per field of view on our mobile microscope), a high mutation detection sensitivity (<1%) can potentially be achieved that is comparable to FDA-approved PCR-based *KRAS* diagnostic tests (the therascreen KRAS RGQ PCR Kit, https://www.qiagen.com/us/resources/technologies/oncology-companion-diagnostics/therascreen-kras-test-usa-labs/#performance).

Next, we validated the targeted sample preparation and sequencing scheme (Fig. 1e) on genomic DNA extracted from cell lines. Sequencing the second base of codon 12 in genomic DNA extracted from A427 cells (heterozygous for a codon 12 mutation) with our mobile phone microscope resulted in 52% Cy3- and 48% Cy5-stained RCPs, well representing the expected ratio of mutant and wild-type molecules (Fig. 2f,g). Mobile sequencing of genomic DNA from Onco-DG1 cells (homozygous KRAS wild type) resulted in predominantly wild-type-specific sequencing signals (Fig. 2g). Regular benchtop fluorescence microscopy, combined with a custom-developed sequencing analysis pipeline^16^ (Supplementary Software 1c), generated sequencing results (Supplementary Fig. 3) that are very well consistent with our mobile-phone-based measurements, demonstrating the utility of our mobile targeted DNA sequencing approach. We further sequenced the extracted DNA from three different colon cancer biopsies. All three tumour samples were measured *KRAS* wild type through our mobile-phone-based targeted sequencing (Fig. 2g), also confirmed by both regular microscopy-based sequencing analysis (Supplementary Fig. 4) and diagnostic PCR analysis (Supplementary Fig. 5).

### Single cell *in situ* analysis can find rare *KRAS* mutant cells

**Figure 3:**
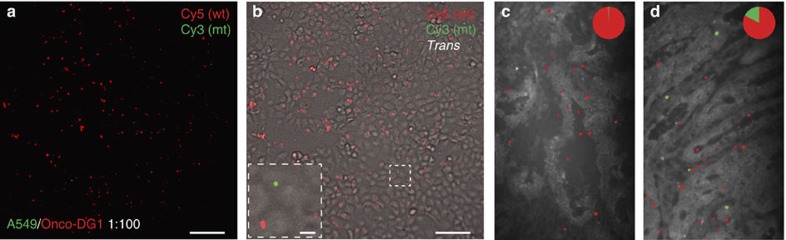
*In situ KRAS* mutation detection in tumour tissues. (**a**,**b**) Cell spike-in imaging experiments: A549 cells carrying a *KRAS* codon 12 mutation were spiked into Onco-DG1 (wild type) cells at a ratio of 1:100. Wild-type RCPs, labelled with Cy5, and mutant RCPs, labelled with Cy3, were imaged using our mobile phone microscope. (**a**) Mobile phone microscope image with Cy5 and Cy3 channels digitally superimposed. (**b**) Mobile phone bright-field image, digitally superimposed with Cy5 and Cy3 channels, visualizes cell boundaries, enabling specific assignments of RCPs to individual cells and identification of *KRAS* mutant cells among wild-type cells. Inset: zoomed-in region from b shows rare *KRAS* mutant cells within the majority of wild-type cells. (**c**,**d**) *In situ KRAS* mutation detection in colon cancer tumour sections. (**c**) Mobile-phone-based fluorescence image of a *KRAS* wild-type tumour section (sample E) and (**d**) a *KRAS* mutant tumour section (sample A), digitally superimposed with base-calling results. Inset pie charts show the ratio between wild type (red) and mutant (green) RCPs in corresponding images. Scale bar, 100 μm (**a**–**d**); 10 μm (**b**, inset).

An alternative mutation analysis scheme, without the need for DNA extraction and sequencing, is genotyping directly *in situ* within tumour tissue sections. Padlock probes, combined with RCA, enable *in situ* analysis of point mutations directly in preserved cells and tissues, adding molecular information to tissue morphology^13^,^17^. These *in situ* generated RCPs can be imaged and quantified using the presented mobile-phone-based multimodal microscopy platform (Figs 1h and 3). We also performed an *in situ* padlock probe-based single base discrimination assay (Fig. 1g) to target the most prevalent mutations in *KRAS* codon 12 and 13 (ref. 17) occurring in 30–40% of human colon cancers^1^,^2^. The multiplex padlock probe panel was first validated on cell lines for high mutant–wild type discrimination of the most recurrent *KRAS* codon 12 and 13 alleles (Supplementary Fig. 6). *In situ* RCA conditions were then optimized for mobile phone microscopy-based imaging with an optimal RCP intensity after a reaction time of 120 min (Supplementary Fig. 7). To quantify RCPs using our mobile phone microscope, we also developed a machine-learning-based image analysis algorithm that permits automated RCP recognition and digital counting (see Methods, and Supplementary Software 2). In total, 14 recognition features (including for example, mean, maximum and minimum intensities) were extracted from individual RCPs in the training set, which included 1,136 RCPs in total. Using this machine learning and training process, an average detection accuracy of 94.9±4.5% was achieved using our mobile phone microscope images, compared to 96.9±1.8% using the images obtained through a regular benchtop microscope.

Next, we tested the utility of this mobile microscope combined with the *in situ* genotyping assay to detect rare cancer cells within a high background of normal cells through a cell spike-in experiment. Cells from the A549 cell line, carrying a mutation in *KRAS* codon 12, were spiked into a background of *KRAS* wild type Onco-DG1 cells in ratios of 1:100 and 1:1,000 (Fig. 3a,b and Supplementary Fig. 8). After the *in situ* padlock assay, the cells were imaged with the mobile phone microscope and the acquired images were processed as described above. *KRAS* mutant cells were detected in low ratios of 1:1,000, yielding a mutant:wild type RCP ratio of 0.49%, compared to 0.15% measured in *KRAS* wild-type cells only (Supplementary Fig. 8b). These data match well with the results obtained using a conventional benchtop microscope (Supplementary Fig. 8b), demonstrating the utility of our mobile platform to identify cancer cells with oncogene mutations among the majority of wild-type cells.

### Tissue *in situ* analysis accurately scores tumour samples

Finally, we tested the clinical applicability of our approach for detecting *KRAS* mutations directly in colon tumour tissue sections (Fig. 3c,d). Our mobile-phone-based multimodal microscope delivers dual-colour fluorescence images that very well match with conventional microscope images of the same samples as illustrated in Supplementary Fig. 9. We imaged several randomly chosen areas on 6 different colon cancer tumour samples (Supplementary Fig. 10) and quantified RCPs (Supplementary Software 3), based on which the genotype is determined (Table 1). Our results revealed that mobile phone microscope-based *in situ* genotyping resulted in 100% concordance to clinical NGS analysis (Table 1) and whole tissue scanning with an automated benchtop fluorescence microscop*e* (Supplementary Table 1). Using mobile-phone-based *in situ* genotyping, a minimum of six images of randomly chosen areas were required to detect a sufficient number of mutant RCPs (Supplementary Fig. 11; Supplementary Note 2) and score all the mutant samples in concordance with clinical NGS data and whole tissue scanning (Table 1; Supplementary Table 1). These results provide evidence that our mobile phone multimodal microscopy platform can be used to analyse RCA-based *in situ* genotyping assays and accurately genotype cancer patient biopsies directly *in situ*, which may facilitate integrating molecular diagnostics with tumour morphology directly in pathologists' offices and POC.

## Discussion

The sample processing steps in our approach are relatively simple to perform and do not require advanced equipment or infrastructure. Yet, in manual operation, preparation of reaction mixes and pipetting steps require a lab technician. The bottleneck in molecular pathology, however, is usually not the availability of lab technicians, but of pathologists. Nevertheless, further automation may facilitate broader implementation and use of our approach in clinical settings. To explore the initial feasibility of integration with micro-fluidics, we also imaged *in situ* RCA assays using the mobile phone microscope directly through a flow cell attached to a sample slide (Supplementary Fig. 12), which showed quite promising results, suggesting that liquid handling can also be integrated onto the same mobile-phone-based interface. However, preparation of tumour cryo-sections, as used in our work, still requires skilled personnel and infrastructure, which may not be available in resource-limited settings. As an alternative, tumour touch imprints can be used as a simple method to prepare samples at POC, especially in resource-limited environments^18^. In this rapid procedure, tumours are gently pressed onto a microscope glass slide leaving a layer of cells attached to the surface with partially preserved morphological structure^18^. These tissue fragments can then be subjected to the point mutation assay^17^ and quantified using our mobile platform.

The sensitivity of our mobile-phone-based targeted sequencing method for extracted tumour DNA is currently limited by the sequencing depth and accuracy to ∼5% mutant:wild-type ratio, which is similar to diagnostic *KRAS* PCR Kits (the therascreen KRAS RGQ PCR Kit, https://www.qiagen.com/us/resources/technologies/oncology-companion-diagnostics/therascreen-kras-test-usa-labs/#performance). Our sequencing depth (currently 100–200 ×) can be increased by using higher DNA concentrations. Higher accuracy can be achieved by adding a third fluorescent channel on our mobile microscope and perform a common RCP anchor stain, which aids in discarding auto-fluorescent objects (Supplementary Figs 3 and 4) that currently contribute to nonspecific signals and limit the accuracy. Ultimately, sequencing several bases will increase our accuracy by discarding auto-fluorescent objects that do not change colour over sequencing cycles.

In conclusion, mobile-phone-enabled molecular diagnostic analysis may provide a simple, cost-effective and yet powerful means to integrate molecular marker information with traditional morphology analysis and might further help digital molecular pathology become widely accessible at POC offices and even in resource-limited settings. The impact of this approach goes beyond molecular pathology. Other important applications may include for example, infectious disease diagnostics, where pathogen identity and load, as well as antibiotic resistance markers, can potentially be measured using the same mobile platform. With a simple DNA sequencing library preparation scheme and the capability to image NGS reactions, mobile-phone-enabled imaging and sensing tools may soon be used for targeted DNA sequencing in clinical settings and POC offices, with the potential to dramatically decrease the cost of NGS-based diagnostics globally.

## Methods

### Ethical permission on human samples

The *in situ* somatic mutation analyses of cancer and patient-matched normal tissues were approved by the Regional Ethical Review Board of Uppsala (2007/116). The tumour biopsy material used for targeted sequencing analysis and basic clinicopathological data were anonymously provided by pathologists without any patient identity or related information, and do not require ethical permission, since only research with biological material that can be traced to a person is subject to ethical approval (The Swedish ethical review act; 2003:460, section 4;3).

### Design of the multimodal cellphone-based microscope

A handheld cellphone-based multimodal microscope was developed by integrating two battery-powered laser diodes (532 nm at 75 mW, Z-Bolt, and 638 nm at 180 mW, Mitsubishi Electric) into a 3D printed optomechanical attachment. Both of these laser diodes were mounted on a focusing stage, each with a tilted incidence angle of ∼75° creating a strong background rejection suitable for dark-field imaging and their illumination spots coincided on the sample plane. The fluorescence signals of the samples were collected by an external lens (focal length: 2.6 mm, UCTronics) added in front of the cellphone camera module and passed through a dual-band emission filter (577/690 nm, Semrock) before reaching the cellphone CMOS image sensor. Using the same cellphone-based microscope design, bright-field images of the samples can also be obtained by illuminating the sample slide vertically with a white LED (897-1183-ND, DigiKey) that was mounted above the sample. All the light sources were powered by a rechargeable battery pack (3.7 V, 1,700 mAh, Vivitar).

The 3D movable sample stage consisted of two moving parts (that is, red and blue parts as shown in Supplementary Fig. 1a) which were also created by 3D printing. The red piece was connected to the basement of the mobile phone attachment via a miniature dovetail stage (DT12, Thorlabs), and served as the z-stage, moving the sample slide in the z direction for focusing adjustment. The lateral movement of the sample slide (*x* and *y* directions) was controlled by the blue part which was connected to the red piece via a 2-axis dovetail translation stage (DT12XY, Thorlabs).

A Nokia Lumia 1020 mobile phone was used in our prototype. It is equipped with a 1/1.5 inch camera sensor that provides 38 megapixels per image (7,152 × 5,368, 4:3 mode) with a pixel size of ∼1.1 μm. The camera also has a long focal length of 6.86 mm and together with the external lens that we have used in our microscope design, it provides an effective magnification factor of ∼2.6X.

### Mobile-phone-based image acquisition

All the three illumination sources (532 nm laser diode, 638 nm laser diode, and white LED) of the mobile phone microscope can be turned on and off independently. Each laser diode provides an oblique illumination angle (∼75°) with their illumination spots coinciding on the focal plane of the mobile microscope. Multicolour imaging was achieved by switching one laser diode on at a time, and fluorescent signals of the samples were separated from the excitation by using a multi-band fluorescence emission filter. For imaging of individual RCPs, a sample slide of interest was inserted into the mobile device from the side. First, the white LED was turned on to align and bring the sample slide into focus using the bright-field imaging mode. Then, 532 and 638 nm laser diodes were turned on sequentially and multiple cellphone fluorescence images (*N*≥5) of the same ROI at each fluorescent channel were captured. All the mobile phone images were recorded in a lossless digital negative (DNG) format by using the default smartphone camera application (that is, Nokia Pro Cam) with the same settings (focusing, white balance, ISO and integration time: 4 s per frame).

### Digital counting of RCPs using machine learning

A machine-learning-based RCP counting algorithm was developed to process the acquired mobile phone microscope images (Supplementary Software 2). The image processing starts with the conversion of the acquired DNG images into 16-bit single-channel TIFF images (using the green channel for Cy3 staining and the red channel for Cy5 staining, respectively). Next, these cellphone image frames of the same ROIs (N≥5) are averaged for both Cy3 and Cy5 channels in order to improve the signal-to-noise ratio of the fluorescent images. The background noise of the averaged images is further reduced by Gaussian filtering. Finally, the Cy3, Cy5, and the transmission/bright-field images of the mobile phone microscope are co-registered and aligned with respect to each other using common spatial features to create digitally superimposed multimodal images. Our machine learning algorithm utilizes a random forest approach to differentiate real RCP signals from background noise, which combines ‘bagging' strategy and the randomness of the features to reduce overfitting. The training libraries for machine learning were established by extraction of characteristic image features from individually validated RCPs using a benchtop fluorescence microscope (Leica TCS SP5, HCX PL APO CS × 40 objective, NA=1.25, oil). A total of 14 different image features including for example, mean, maximum and minimum intensities of individual RCPs were extracted from 20 Cy3 channel images and 20 Cy5 channel images, respectively, and used as a training set for blind counting and analysis of newly acquired Cy3 and/or Cy5 mobile phone microscope images in future mobile microscopy experiments. The gold standard RCP counts used in our comparison were obtained from benchtop microscope images (for example, Supplementary Fig. 8a) by using an intensity threshold-based automated algorithm. While this automated counting in the current study was done using a desktop computer, for both the benchtop and mobile-phone-based microscope images, it is also possible to conduct the same analysis on the smartphone itself using a custom-written application^19^.

### On-slide RCA

Biotinylated circles were prepared by mixing 500 pM biotinylated ligation template, 100 pM padlock probe (Supplementary Table 2), 0.2 mg ml^−1^ BSA (NEB), 1 × phi29 polymerase reaction buffer (33 mM Tris-acetate (pH 7.9), 10 mM Mg-acetate, 66 mM K-acetate, 0.1% (v/v) Tween 20, 1 mM DTT, Thermo Scientific), 680 nM ATP and 20 mU μl^−1^ T4 ligase in a final volume of 25 μl. The mix was incubated for 15 min at 37 °C, followed by inactivation of the enzyme at 65 °C for 2 min. Circles were diluted 1:10 from 100 pM to 1 fM in binding buffer (10 mM Tris-HCl pH 8, 10 mM EDTA, 0.05% Tween 20, 1 M NaCl). 50 μl secure seals (Grace Biolabs) were mounted on Neutravidin glass slides (Poly-An). 50 μl circle dilutions in duplicates from 10 pM to 1 fM were added and incubated for 3 h at room temperature, followed by two washes with PBS+Tween 0.05% (PBS-T).

Rolling circle amplification was performed on slide by adding RCA reaction mix, containing 0.2 mg ml^−1^ BSA, 1x phi29 polymerase reaction buffer (33 mM Tris-acetate (pH 7.9), 10 mM Mg-acetate, 66 mM K-acetate, 0.1% Tween-20 (v/v), 125 mM dNTPs (DNA Gdansk) and 500 mU μl^−1^ phi29 polymerase (Olink Biosciences). The reaction was incubated overnight at room temperature. After washing twice with PBS-T, RCA products were fluorescently labelled by addition of 100 nM Cy3 and 100 nM Cy5-modified detection oligonucleotides (Supplementary Table 2) in hybridization buffer (2 × SSC, 20% Formamide) and incubation for 1 h at room temperature. After three washes with PBS-T the secure seals were detached from the slide, followed by an ethanol series (70% 1′, 80% 1′, 100% 1′). The slides were mounted in SlowFade Gold Antifade (Thermo scientific) mounting medium with a 25 × 60 mm coverslip (Themo Scientific). For quantification, three randomly chosen positions per duplicate were imaged with the mobile phone microscope and a regular epifluorescence microscope (Zeiss Axio Imager Z2) using a 20 × microscope objective. Finally, images were analysed through CellProfiler 2.1.2 (Broad Institute) software with an in-house script for RCP recognition and quantification (Supplementary Software 1a).

### Targeted sequencing of *KRAS* codon 12 and 13

To prepare surfaces for sequencing reactions, Neutravidin modified slides (Polyan, Germany) were functionalized with a dense layer of biotinylated selector probes (Supplementary Table 2) by incubation in 200 μl secure seal chambers (Grace Biolabs) in binding buffer containing 1 μM biotinylated selector probe (Supplementary Table 2), 10 mM Tris-HCl pH7.5, 5 mM EDTA, 0.1% Tween-20 and 1 M NaCl for 20 min. The mix was removed and the slide washed with PBS (0.05% Tween-20) twice. The secure seal chamber was removed. An in-house PDMS fabricated mini-well gasket with ∼2 mm^2^ area wells was applied on top of the slide and clamped so that the micro-well chambers seal on top of the slide. The mini wells serve as confined reaction chambers for the parallel processing of several samples on one slide, which is optional to increase throughput of analysis.

Sequencing libraries were prepared from genomic DNA isolated from cell lines and from colon cancer tumour biopsies by column-based DNA purification (Mini-preparation Kit, Qiagen, Germany). For that purpose approximately 5 × 10^6^ cells were centrifuged down and the pellet resolved in lysis buffer. Tumour DNA was extracted from 10–20 mg tumour biopsy material. Approximately 100 ng μl^−1^ DNA concentrations were obtained. 500 ng DNA was subjected to restriction digestion in NEB cutsmart buffer (50 mM Potassium Acetate, 20 mM Tris-acetate, 10 mM Magnesium Acetate), 300 μg ml^−1^ BSA and 0.25 U μl^−1^ MseI at 37 °C for 1 h. Before application to the selector probe functionalized slides, DNA was heat denaturated at 100 °C for 10 min and immediately put on ice. DNA samples were mixed with Ampligase buffer (20 mM Tris-HCl pH 8.3, 250 mM KCl, 100 mM MgCl_2_, 5 mM NAD, and 0.1% Triton X-100), 0.2 mg ml^−1^ BSA (NEB) and 250 mU μl^−1^ Ampligase (Epicentre) to a final volume of 20 μl. The samples were then added onto the selector probe functionalized slides in the mini wells, prepared as described above. The wells were sealed and the reaction incubated overnight at 45 °C. During the ligation reaction the genomic MseI *KRAS* fragments diffuse to the slide surface, the fragment ends hybridize onto the selector probes and are ligated (Fig. 1e, for illustration). The reaction mixtures were removed and the mini wells were detached. 200 μl secure seal reaction chambers were mounted on top of the area, on which the samples were spotted with help of the mini wells. The continuous steps were then performed in 200 μl chambers to process all the spotted samples in parallel from there on. The spotted samples were washed twice in PBS-Tween 0.05%. The ligated DNA fragments were amplified by RCA through addition of RCA reaction mixture, as described above, with addition of 25 nM *KRAS* specific compaction oligonucleotide^21^. The reaction was incubated overnight at room temperature and washed twice with PBS-Tween 0.05%.

Sequencing by ligation reactions were performed by first hybridizing a common anchor probe to RCA products by addition of 100 nM Alexa750 labelled anchor probe (Supplementary Table 2) in hybridization buffer (2 × SSC, 20% Formamide) and incubation for 1 h at room temperature. After two washes in hybridization buffer without anchor probe and 1 wash in PBS-Tween 0.05%, sequencing reaction mix was applied, containing fluorescently labelled 9mer oligonucleotides with degenerated base composition on all positions except for the position that was sequenced (Supplementary Table 2). On the query position nucleotide A was coded by fluorescent Cy5 label, T by FITC, G by Cy3 and G by Texas red (or blank). Sequencing reaction mix contained 1 × phi29 reaction buffer, 250 nM ATP (Fermentas), 0.2 mg ml^−1^ BSA, 100 nM sequencing library each and 100 mU μl^−1^ T4 ligase (DNA GDansk). The reaction was incubated at room temperature (22 °C) for 1 h. The reaction was stopped by removing the reaction mix and washing three times in PBS-Tween 0.05%.

To determine the sequenced nucleotide, the reactions were fluorescently imaged as described below. To sequence the next nucleotide position, the ligated sequencing library and anchor probe are enzymatically removed through UNG treatment, which removes the Uracils in the anchor probe and weakens the hybridization. For that purpose 20 mU μl^−1^ UNG (Fermentas) in phi29 reaction buffer and 0.2 mg ml^−1^ BSA were applied and incubated at room temperature for 10 min. The mixture was removed, the slides washed in PBS-Tween 0.05% twice and then incubated in hybridization buffer (2 × SSC, 20% Formamide) for 20 min at 45 °C. The slides were washed twice in PBS-Tween 0.05%. After that the sequencing cycle is repeated by hybridization of the same anchor probe and addition of sequencing reaction mix containing sequencing library probes that interrogate the second nucleotide position.

To image the sequencing reactions the secure seals were detached from the slide, followed by an ethanol series (70% 1′, 80% 1′, 100% 1′). The slides were then mounted in SlowFade Gold mounting medium with coverslip covering the sample area. Mobile-phone-based microscopic imaging was performed as described above. Initially, to validate that sequencing reactions were successful, the reactions were imaged on Zeiss Axio Imager Z2 by fluorescent imaging of sequenced RCPs in Alexa 750, Cy3, Cy5, FITC and Texas red channels using a × 20 microscope objective. Alexa750 stain commonly identifies all RCPs and helps discarding non-RCPs that are not labelled with Alexa750. Mobile-phone-based microscopic imaging was performed with Cy5 and Cy3 excitation sources as detailed above. Sequencing reactions were quantified in an imaging analysis pipeline with Cell Profiler 2.1.2 (Broad Institute), ImageJ and Matlab (Mathworks) software, using a customized version of an analysis pipeline reported previously^16^ (Pipeline and scripts available in Supplementary Software 1b,c, for mobile images and benchtop images, respectively). Double-stained fluorescent signals, with a ratio that is higher than 0.3, were discarded as auto-fluorescent objects.

### Cell line and tumour section sample preparations

ONCO-DG-1 and A-427 cell lines were cultured in RPMI culture medium (Sigma) without L-Glutamine supplemented with 10% FBS (Sigma) 2 mM L-Glutamine (Sigma) and 1 × Penicillin-Streptomycin (PEST, Sigma). A-549 was cultured in DMEM (Sigma) supplemented 10% FBS and 1 × PEST. When confluent, all cell lines were seeded on Superfrost Plus slides (Thermo Scientific) and allowed to attach for 12 h. The cells were then fixed in 3% paraformaldehyde (Sigma) in DEPC-treated PBS (DEPC-PBS) for 15 min at room temperature. After fixation, slides were washed twice in DEPC-PBS and dehydrated through an ethanol series of 70, 85 and 100% for 4 min each. Secure seal chambers were mounted on the slides, the cells were hydrated by a brief wash with PBS-T (DEPC-PBS with 0.05% Tween 20 (Sigma)) followed by a permeabilization with 0.1 HCl in H_2_O for 1 min at room temperature. All cell lines were obtained from DSMZ, and were tested negative for mycoplasma infection. According to the International Cell Line Authentication Committee, the Onco-DG1 cell line may be contaminated with OVCAR-3, an ovarian cancer cell line. We used Onco-DG1 in our work, because it has an elevated expression of wild-type *KRAS*. A potential contamination with OVCAR-3, likewise *KRAS* wild type, would not have affected our results and conclusions.

Sensitivity and specificity of the *KRAS* mutation detection assay were validated in cell line dilution experiments. To test for the lowest detectable ratio of mutant cells in a majority of wild-type cells a spike-in experiment was performed. A-549 (*KRAS* mutant G12S) cells were spiked into Onco-DG1 *KRAS* wild-type cells in ratio 1:100 and 1:1,000. Cell mixtures were seeded on Superfrost Plus slides and left to attach for 12 h. The slides were washed in cold PBS and fixed in 3% PFA for 15 min and further processed as described above.

Fresh frozen human tumour tissues from colorectal cancer patients were anonymously obtained from Biobank Uppsala, Sweden, without a link to any patient related information. The somatic mutation analyses of cancer and patient-matched normal tissues were approved by the Regional Ethical Review Board of Uppsala (2007/116). Tape transfer sections (4 μm thick) were fixed in 3% paraformaldehyde in DEPC-PBS for 45 min at room temperature followed by washing with DEPC-PBS. The samples were then treated with 0.01% pepsin (Sigma) in 0.1 M HCl at 37% for 90 s. The digestion was stopped by two washes in DEPC-PBS-T for 2 min. Slides were then dehydrated through an ethanol series of 70, 85 and 100% for 2 min each.

### *In situ* mutation detection assay and tumour genotyping

All *in situ* reactions were performed in Secure-Seal Chambers (Grace Bio-Labs Inc.), with 50 μl reaction volumes (size 9 mm diameter, 0.8 mm deep) for cells, and either 100 μl (size 13 diameter, 0.8 mm deep) or 200 μl (size 22 mm diameter, 0.8 deep) for tissues, depending on the size of the tissue sample. Reverse transcription reaction mixture was added to the chambers, containing 1 μm cDNA primer (Supplementary Table 2), 20 U μl^−1^ TranscriptMe reverse transcriptase (DNA Gdansk), 500 μM dNTP (Thermo Scientific), 0.2 mg ml^−1^ BSA (NEB), and 1 U μl^−1^ RiboLock RNase Inhibitor (Thermo Scientific) in TranscriptMe reaction buffer (DNA Gdansk). Cell slides were incubated for 3 h and tissue sections overnight at 45 °C. After brief wash in PBS-T, cells were postfixated in 3% PFA for 10 min and tissues for 30 min at room temperature. The padlock probe assay was performed as follows:^17^ In brief, 100 nM of each padlock probe was added in a mix of 1 U μl^−1^ Ampligase (Epicentre), 0.4 U μl^−1^ RNase H (DNA Gdansk), 1 U μl^−1^ RiboLock RNase Inhibitor, 50 mM KCl, 20% formamide in Ampligase buffer. Incubation was performed first at 37 °C for 30 min, followed by 45 min at 45 °C. After ligation, slides were washed by flushing the chambers with PBS-T. After a brief wash in PBS-T, RCA was performed, in a mix containing 1 U μl^−1^ phi29 DNA polymerase (Olink Biosciences) in phi29 buffer (Thermo Scientific), 250 μM dNTP, 0.2 mg ml^−1^ BSA and 5% Glycerol. RCA was performed on cell slides at 37 °C for 3 h (30, 60, 120 and 300 min during RCA time course experiment). Tissue samples were incubated overnight at room temperature. After RCA, the slides were washed with PBS-T. RCPs were visualized by hybridizing 0.5 μM of each corresponding detection probe (Supplementary Table 2) in 2 × SSC and 20% formamide (Sigma) for 30 min at 37 °C. Cell nuclei were stained with DAPI during the same step. Secure-seals were removed and the slides were dehydrated through an ethanol series of 70, 85 and 100% for 2 min each. Slides were then mounted using Slowfade Antifade reagent (Life Technologies). Samples were imaged with our mobile phone microscope as described earlier. For comparison, the same samples were also imaged with Zeiss Axio Imager Z2 using a × 20 objective lens with fluorescent channels for DAPI (cell nuclei), Cy3 (mutant specific RCPs), Cy5 (wild-type-specific RCPs), and Alexa750 (Actb specific RCPs).

Mobile-phone-based microscopy images of cell line dilution experiments were analysed and RCPs quantified as described above. For tumour section analysis with mobile phone microscopy, several randomly chosen regions and some predefined regions were imaged. tumour sections were additionally scanned in its full size using regular benchtop microscopy with an automated scanning stage (Zeiss Axio imager Z2). RCPs in both mobile phone and regular microscope images were analysed using the open source software CellProfiler (Pipeline available in Supplementary Software 3). RCPs were identified and quantified using different thresholds according to the intensity of the RCPs and the auto-fluorescence level of the tissue section. Images were filtered using the ‘Enhance of Suppress Features' module and RCPs then identified with the ‘Identify Objects' module using size thresholds of 2–8 pixels and intensity thresholds of 0.1–0.4, depending on the sample and the level of auto-fluorescent structures. Double stained auto-fluorescent structures in RCP size and intensity range were identified and filtered out using the Image Math module in CellProfiler. Finally, wild type and mutant specific RCPs were counted and replotted onto the tumour section image. Ratios of mutant*/*wild type RCPs per individual ROI and for all the ROIs combined were determined. The threshold for scoring a tumour section as wild type was set at 8%, under which tumour sections were scored as wild type, and above which tumour sections were scored as mutant. The 8% threshold is based on the clinically scored wild-type section that resulted in the highest mutant/wild type ratio (tumour section E1: 7.8% on whole tissue scanning). This background arises from strong auto-fluorescent structures, especially in necrotic regions, that can contribute to elevated unspecific mutant RCP counts.

### Data availability

Data can be obtained by request from the authors. Image analysis codes and pipelines are available in the Supplementary Software.

## Additional information

**How to cite this article:** Kühnemund, M. *et al*. Targeted DNA sequencing and *in situ* mutation analysis using mobile phone microscopy. *Nat. Commun.* **8,** 13913 doi: 10.1038/ncomms13913 (2017).

**Publisher's note**: Springer Nature remains neutral with regard to jurisdictional claims in published maps and institutional affiliations.

## Supplementary Material

Supplementary Information Supplementary Figures, Supplementary Tables, Supplementary Notes, and Supplementary References

Supplementary Software Image analysis software folder, containing Matlab codes and Cell profiler analysis pipelines.

## Figures and Tables

**Table 1:** Mobile-phone-based *in situ KRAS* mutation analysis of colon tumour samples.

**Tumour sample**	**No. of ROIs**	**No. of MT RCPs**	**No. of WT RCPs**	**Ratio MT/WT**	**Score**	**Clinical NGS**
A	14	82	345	23.8%	Mutant	G-12S 18%
E	9	3	546	0.6%	Wild type	Wild type
F	7	50	262	19.1%	Mutant	G-12V 37%
I	8	140	255	54.9%	Mutant	G-12V (ratio NA)
E1	6	51	706	7.2%	Wild type	Wild type
J	6	64	857	7.5%	Wild type	Wild type

MT, mutant; RCPs, rolling circle amplification products; ROIs, region of interests; NA, not applicable; NGS, next-generation sequencing; WT, wild type.

Six different colon cancer tumour samples were subjected to *in situ KRAS* mutation detection assay and several randomly chosen ROIs within the samples were imaged with our mobile phone microscope. *KRAS* mutant and *KRAS* wild-type RCPs were quantified and the corresponding measured ratios and genotyping scores are listed together with the results of NGS-based clinical analysis per tumour sample.
