# Supplementary material for: Targeted DNA sequencing and in situ mutation analysis using mobile phone microscopy
Source: Nat Commun. 2017 Jan 17;8:13913. doi: 10.1038/ncomms13913 (PMC5247573; doi:10.1038/ncomms13913)
Supplement: Supplementary Information — Supplementary Figures, Supplementary Tables, Supplementary Notes, and Supplementary References [file ncomms13913-s1.pdf]

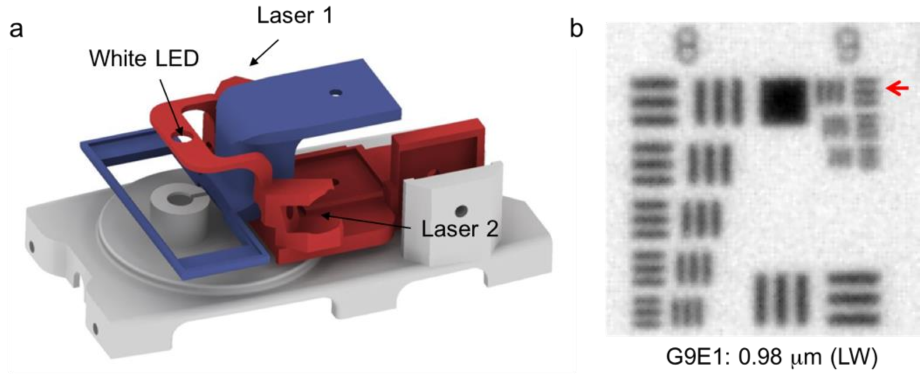

Supplementary Figure 1: (a) 3D illustration of the mobile phone microscopy attachment, containing a 3D movable sample holder (red piece for z movement and blue piece for x-y movement). Illumination sources include two compact laser diodes (at 532 and 638 nm) and a white LED, all battery powered. (b) A bright-field mobile phone based microscopic image of a resolution test target, demonstrating a half-pitch resolution of 0.98 μm (red arrow).

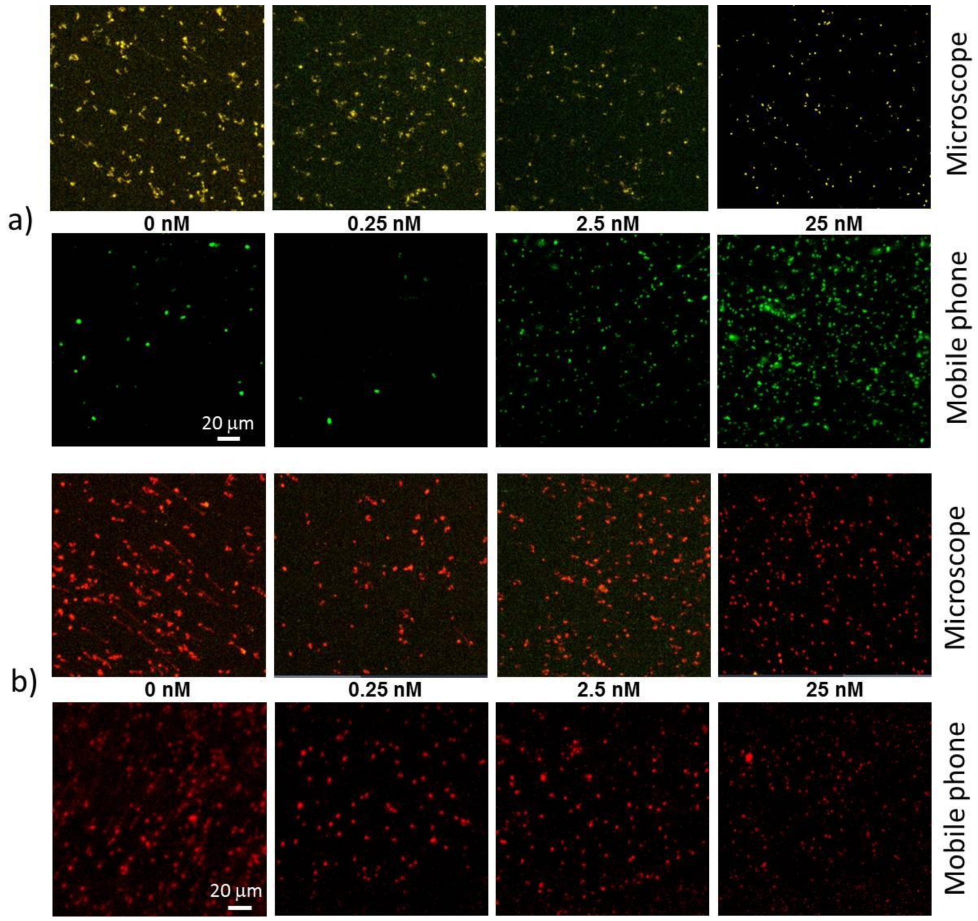

Supplementary Figure 2: Titration of compaction oligonucleotide (CO) concentration for increasing RCP integrity during sequencing reaction. Selector probe circularized synthetic KRAS fragments with mutation at 12.1 (a) or wild type sequence (b) were circularized and amplified through RCA and sequenced by ligation. An increasing amount of compaction oligonucleotide was used, leading to higher RCP integrity.

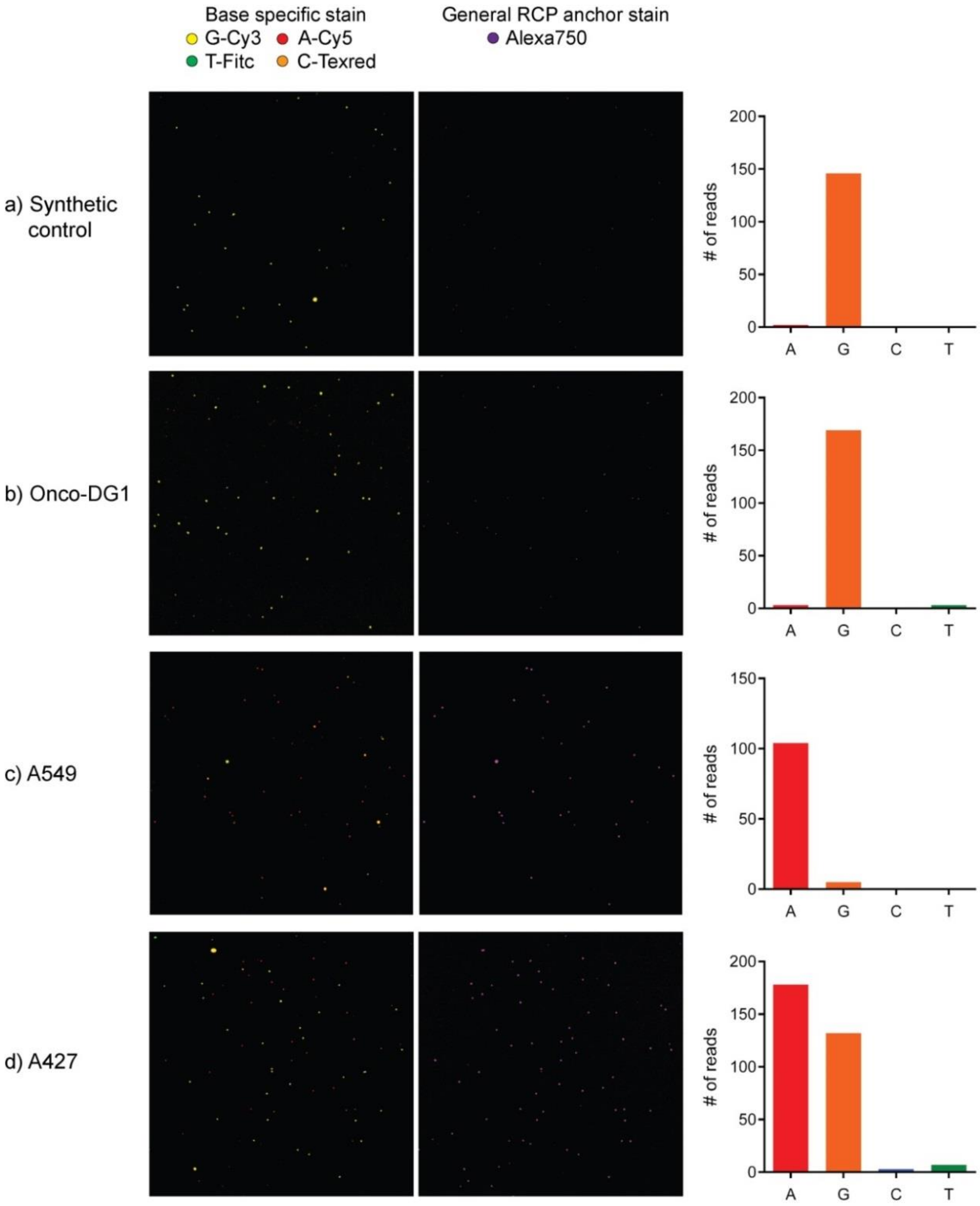

Supplementary Figure 3: Sequencing results of cell line extracts obtained by regular microscopy and our custom-designed image analysis pipeline. (a) 500 zmol synthetic KRAS fragment, (b) Onco-DG1 cell line extracted DNA, (c) A549 cell line extracted DNA, (d) A427 cell line extracted DNA. Left column: Sequencing raw signals in fluorescent channels of Cy3 (corresponding to base specific stain G), Cy5 (corresponding to base specific stain A), FITC (corresponding to base specific stain T) and Texred (corresponding to base specific stain C). The plots on the right column depict the RCP anchor primer labeled with Alexa750, which generally stains all RCPs and can be used as RCP identification segmentation signal. Graphs represent base-calling analysis after sequencing the first 2 bases in codon 12.

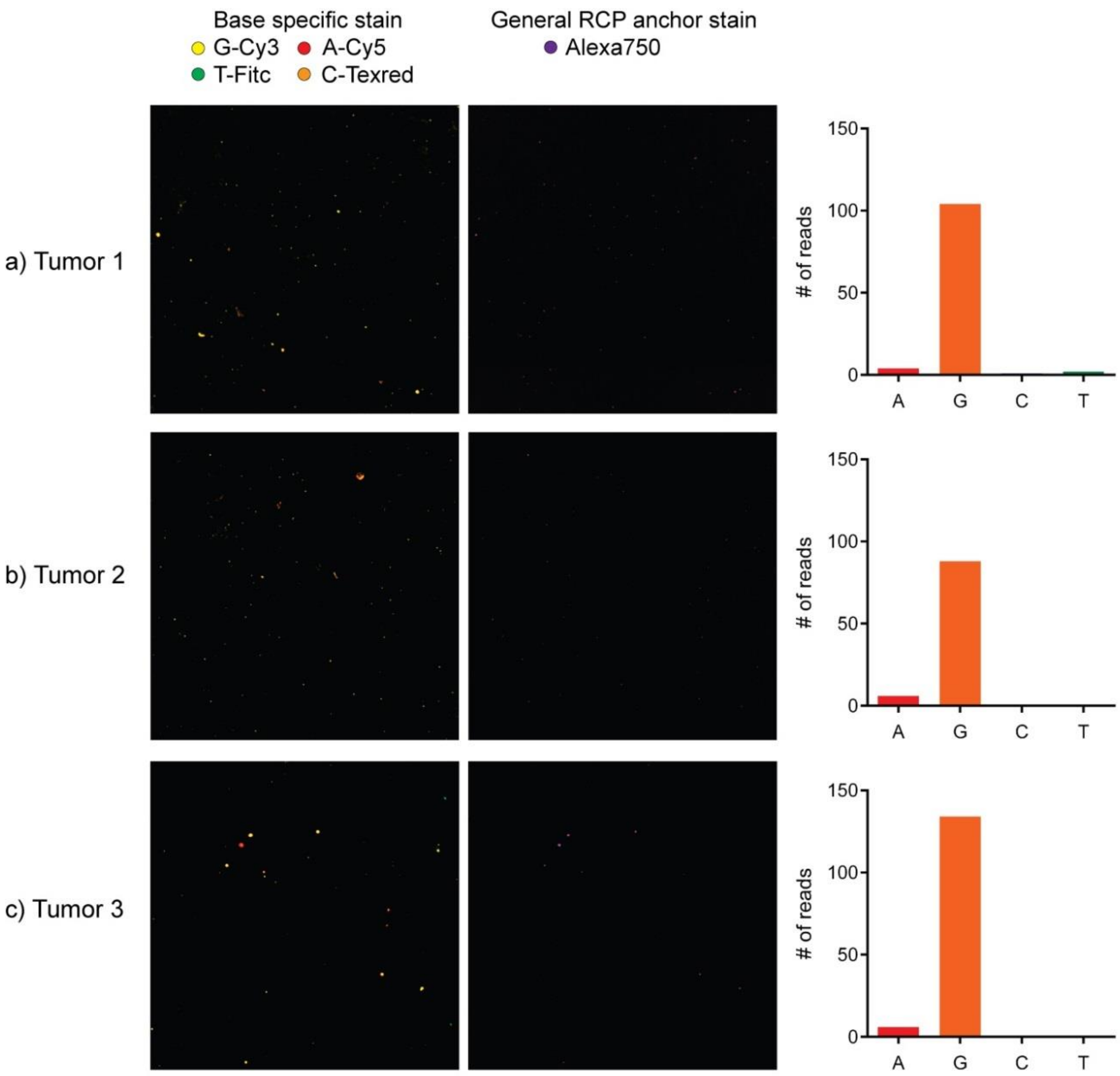

Supplementary Figure 4: Sequencing results of extracted tumor DNA using regular fluorescence microscopy and our custom-designed image analysis pipeline. (a) Extracted DNA from colon tumor 1, (b) colon tumor 2, (c) colon tumor 3. Left column of images: Sequencing raw signals in fluorescent channels Cy3 (corresponding to base specific stain G), Cy5 (corresponding to base specific stain A), FITC (corresponding to base specific stain T) and Texred (corresponding to base specific stain C). The plots on the right column depict the RCP anchor primer labeled with Alexa750, which generally stains all RCPs and can be used as RCP identification segmentation signal. Graphs represent base-calling analysis after sequencing the first 2 bases in codon 12.

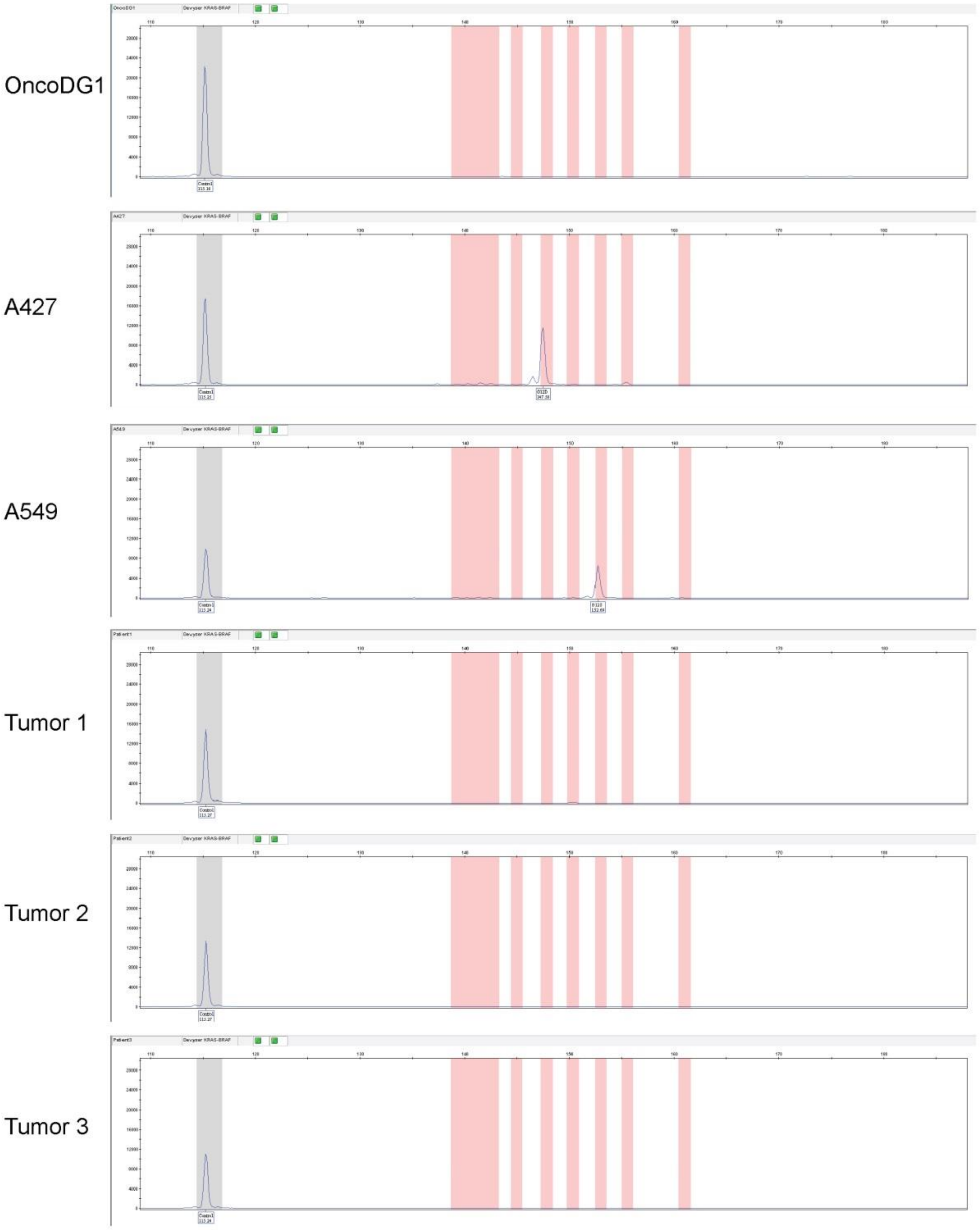

Supplementary Figure 5: KRAS mutation analysis using a diagnostic PCR Kit (Devyser, Sweden) with capillary gel-electrophoresis read-out.

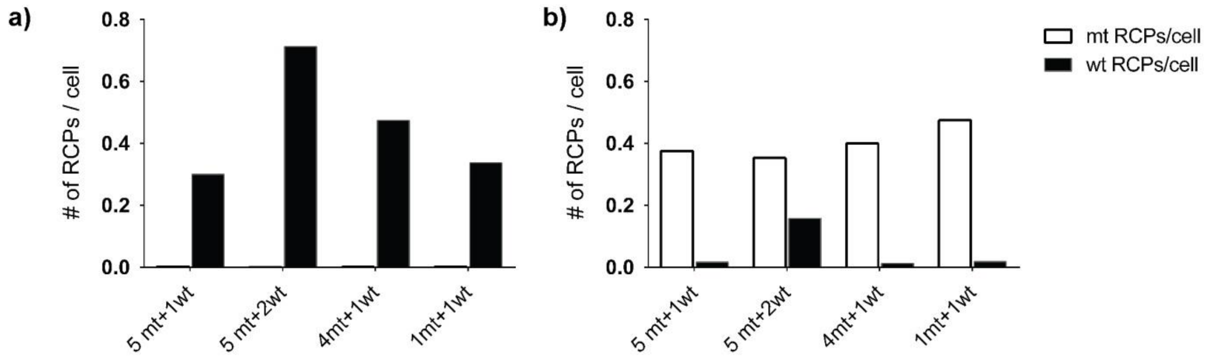

Supplementary Figure 6: Optimization of padlock probe cocktail targeting the most prevalent KRAS point mutations applied to (a) OncoDG1 wild type, and (b) A549 mutant cell lines, and analyzed through regular microscopy. The average number of RCPs per cell is plotted as a function of wild type- and mutant specific probe combinations.

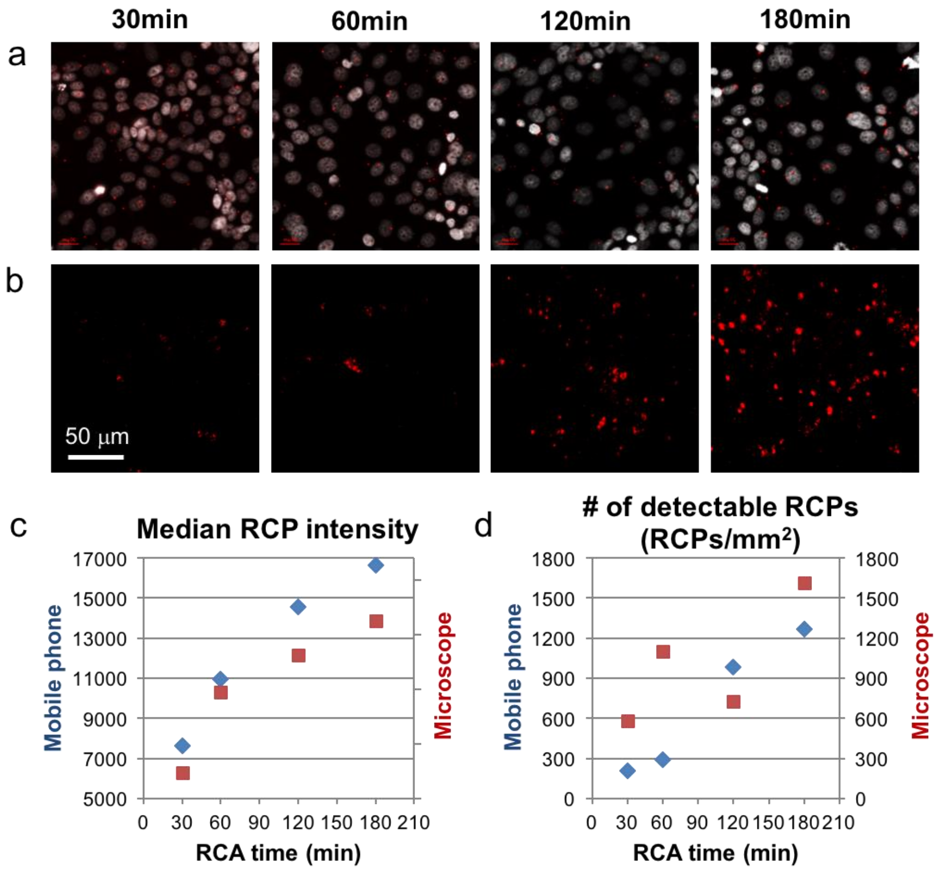

Supplementary Figure 7: Analysis of RCA reaction time course. *In situ* RCA of wild type KRAS probes in Onco-DG1 cells was performed for different durations and imaged through (a) regular benchtop microscopy and (b) mobile phone based microscopy. (c) Median intensity per RCP increases as a function of the RCA time. (d) With increasing RCA time and signal intensity, the number (#) of detectable RCPs per mm^2 also increases over time.

a

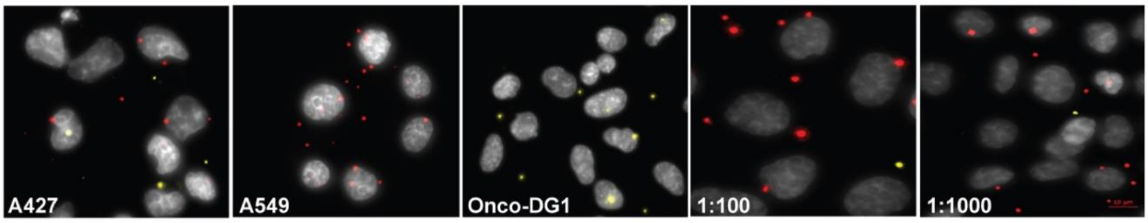

b

Spike-in ratio (A549: Onco-DG1)	Microscope				Cellphone		
	# of Cells	# of MT RCPs Cy3	# of WT RCPs Cy5	Measured ratio (Cy3/Cy5)	# of MT RCPs Cy3	# of WT RCPs Cy5	Measured ratio (Cy3/Cy5)
1:100	8590	153	7118	**2.15%**	65	3352	**1.94%**
1:1000	33115	44	20811	**0.21%**	10	2024	**0.49%**
Only Onco-DG1	3824	3	2259	**0.13%**	12	7746	**0.15%**

Supplementary Figure 8: *In situ* detection of KRAS point mutations in cell lines. Detection of KRAS wild-type (red RCPs) and mutant (yellow RCPs) transcripts in different cell lines, analyzed through benchtop and mobile phone based microscopy. (a) Conventional benchtop microscopy images of the heterozygous A427 cell line (expressing both wild and 12.2. mutant KRAS transcripts), the wild type cell line OncoDG1, the homozygous 12.1 mutant cell line A-549, and A549 cells spiked into Onco-DG1 cells in ratios of 1:100 and 1:1000, respectively. DAPI stained cell nuclei are shown in grey. (b) Summary of the results of these spike-in experiments based on regular benchtop fluorescence microscopy (left) and mobile phone microscopy (right), demonstrating the capability of our mobile microscope to detect < 1% mutant RCPs within a background of wild type RCPs.

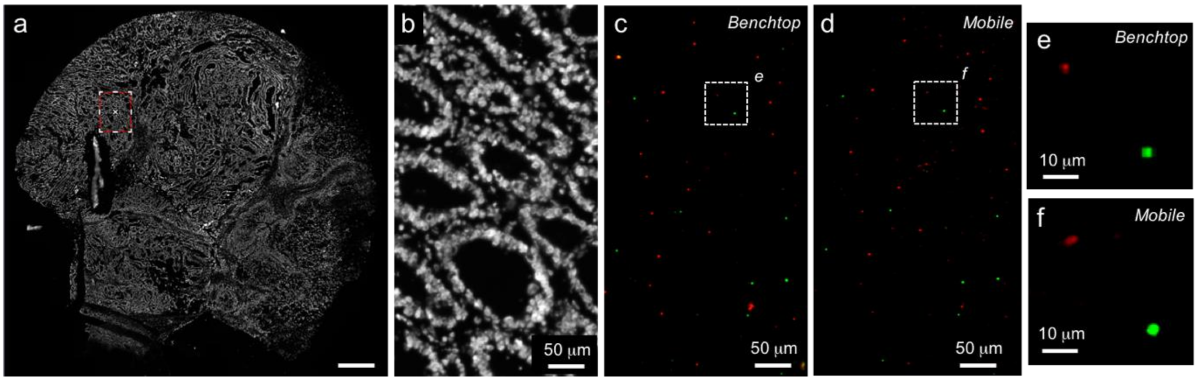

Supplementary Figure 9: *In situ* detection of KRAS mutations in preserved tumor tissue samples. (a) Whole tissue scan of DAPI stained nuclei (grey) using a benchtop microscope, illustrating the tissue morphology. (b) A zoomed-in region of (a). (c) The same region of interest as in (b), imaged with a benchtop microscope, where Cy3 and Cy5 channels are digitally superimposed. (d) is the same regions of interest as (b and c), except captured using our mobile phone microscope. (e,f) Zoomed-in regions taken from (c,d), respectively, demonstrating a very good comparison between conventional benchtop microscopy and our mobile phone-based microscope for the detection of both wild type (red, Cy5) and mutant (green, Cy3) RCPs directly in tumor tissue slices.

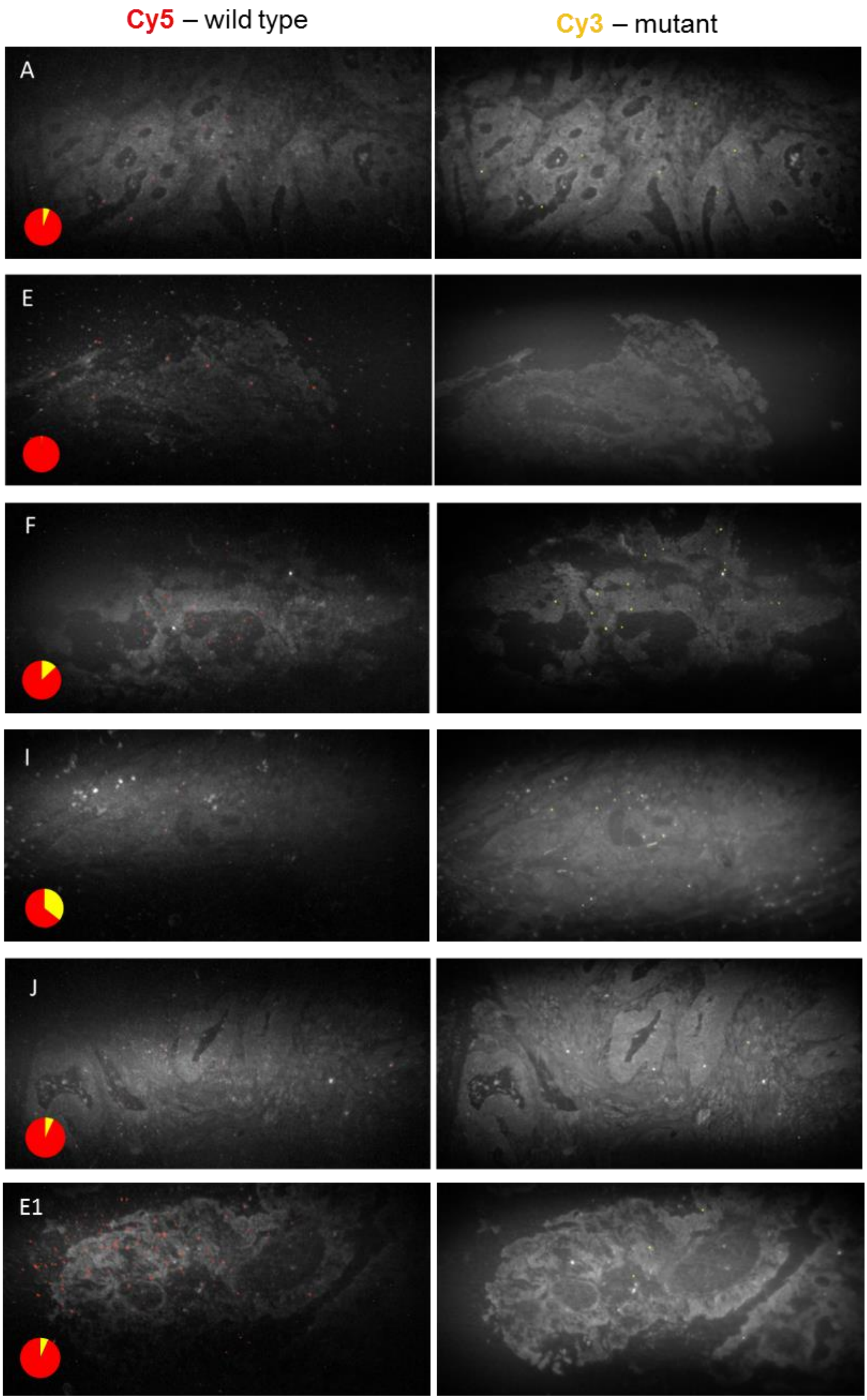

Supplementary Figure 10: Representative mobile phone based multimodal microscope images from each tumor sample. Left column: Mobile phone images acquired with Cy5 filter. KRAS wild type

specific RCPs were identified though an image analysis pipeline (as detailed in our Materials and Methods Section) and digitally superimposed on top of the original fluorescence image captured by the mobile phone microscope. Right column: Mobile phone images acquired with Cy3 filter. KRAS mutant RCPs were identified through image analysis and digitally superimposed on top of the original mobile phone fluorescence image. Note that objects appearing in both channels are discarded as auto-fluorescent objects. Pie chart insets indicate the ratio between wild type (red) and mutant (yellow) RCPs in the represented mobile phone microscope images.

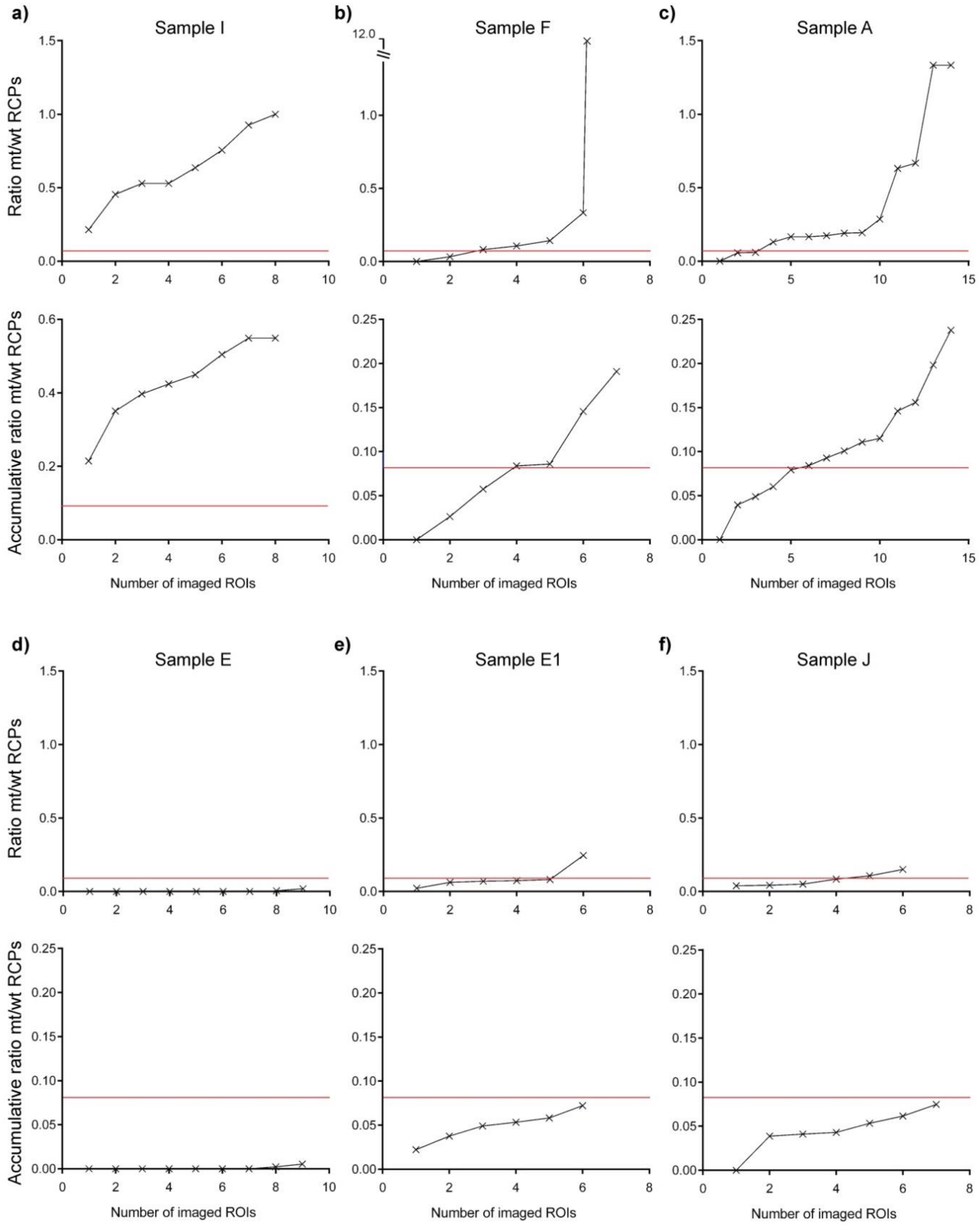

Supplementary Figure 11: Individual and accumulative mutant/wildtype RCP ratios determined by mobile phone based imaging of randomly chosen ROIs in KRAS mutant typed tumor samples I (a), F (b), and A (c), and KRAS wildtype scored tumor samples E (d), E1 (e) and J (f). The ratios obtained from analyzed ROIs are plotted individually (upper row) and accumulatively (lower row), sorted from low to high. In order to detect a sufficient number of mutant signals and correctly genotype the

tumor as mutant (above 8% mutant/wildtype ratio, indicated with the red solid lines) at least 1 randomly chosen ROI has to be imaged in tumor sample I, at least 4 have to be imaged in sample F and at least 6 have to be imaged in sample A.

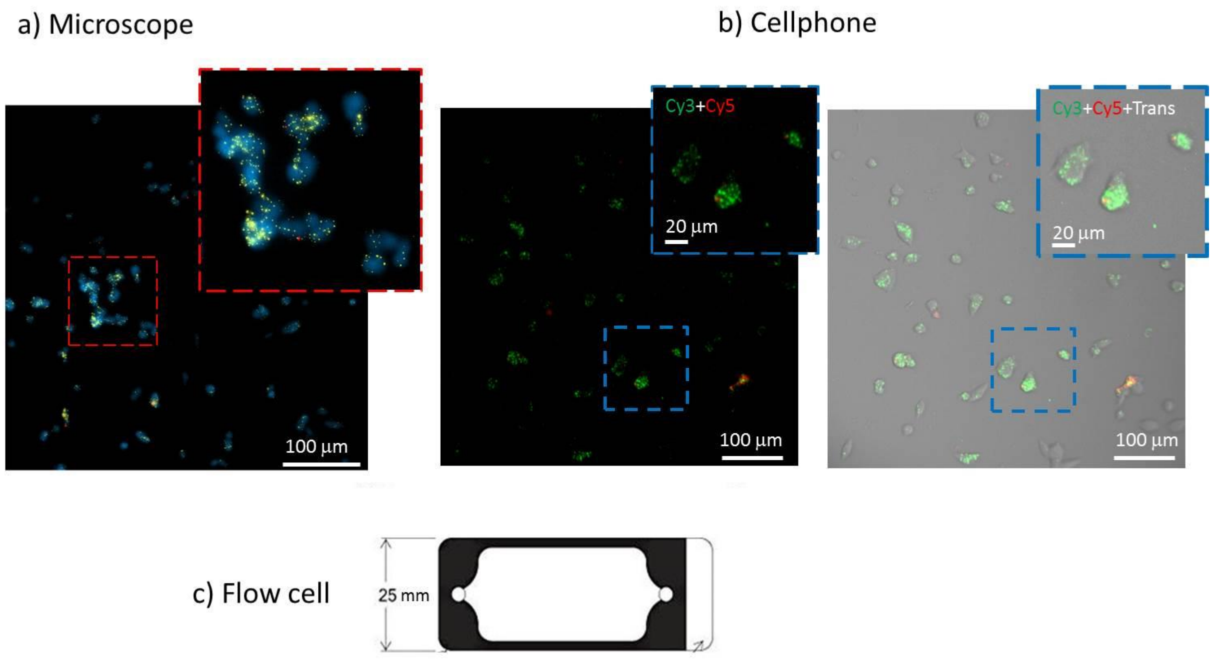

Supplementary Figure 12: Imaging of an *in situ* RCA assay using our mobile phone microscope through a flow cell attached to a sample slide. (a) Cells with Actin beta (Cy3, yellow) and KRAS mutant (Cy5, red) detected transcripts imaged using a benchtop microscope through a flow cell. (b) The same as in (a), but imaged using our mobile phone based microscope directly through the flow cell, where Cy3 stained RCPs are depicted in green, and Cy5 stained RCPs are depicted in red. 'Trans' refers to the transmission mode of the mobile microscope, in addition to the fluorescent channels. (c) Schematics of the microfluidic flow cell (Grace Biolabs, USA) through which various reagents can be added to and removed from the sample through fluidic automation.

Supplementary Table 1: Quantification of 4 of the 6 tumor samples through whole tissue scans using a benchtop microscope.

Tumor sample	# of ROIs	# of RCPs Cy3 mutant	# of RCPs Cy5 wild type	Measured ratio (mutant/wild type)	NGS
A	whole tissue	251	703	**35.7 %**	G-12S 18%
F	whole tissue	550	1054	**52 %**	G12V 37%
I	whole tissue	960	1342	**71.1 %**	G-12V (ratio n.a.)
E1	whole tissue	199	2522	**7.8 %**	wild type

Supplementary Table 2: Oligonucleotides

**Primers**	**Sequences (5´ - 3´)** (+= LNA base)
P-KRAS-c12/13	CC+TC+TA+TT+GT+TGG+AT+CATATTCGTC
P-ACTB	GGCAAAGGCGAGGCT
**Padlock probes**	**Sequences (5´ - 3´), oligos are 5´phosphorylated**
F5_vRNA_padlock	CTGTTTGAAATTTCCCTTTGA AGTCGATAGTCACGGCTACT AGTCGGAAGTACTACTCTCT TGTATGCAGCTCCTCAGT AACCATTGTTCTTTGTGCAG
PP-KRAS-G12S	GTGGCGTAGGCAAGATTCTAGATC*CCTCAATGCACATGTTTGGCTCC*GGTTCAAGTGGTAGTTGGAGCTA
PP-KRAS-G12D	TGGCGTAGGCAAGAGTTCTAGATC*CCTCAATGCACATGTTTGGCTCC*GGTTCAAGGGTAGTTGGAGCTGA
PP-KRAS-G12V	TGGCGTAGGCAAGAGTTCTAGATC*CCTCAATGCACATGTTTGGCTCC*GGTTCAAGGGTAGTTGGAGCTGT
PP-KRAS-G12A	TGGCGTAGGCAAGAGTTCTAGATC*CCTCAATGCACATGTTTGGCTCC*GGTTCAAGGGTAGTTGGAGCTGC
PP-KRAS-G13D	CGTAGGCAAGAGTGCTTCTAGATC*CCTCAATGCACATGTTTGGCTCC*GGTTCAAGAGTTGGAGCTGGTGA
PP-KRAS-wt1	GTGGCGTAGGCAAGATCCTAGTAATC*AGTAGCCGTGACTATCGACT*GGTTCAAAGTGGTAGTTGGAGCTG
PP-KRAS-wt3	CGTAGGCAAGAGTGCTCCTAGTAATC*AGTAGCCGTGACTATCGACT*GGTTCAAAGAGTTGGAGCTGGTGG
PP-ACTB-II	AGCCTCGCCTTTGCCTCCTCTATGATTACTGACTGCGTCTATTTAGTGGAGCCGCATCTATCTTCTTTCGCCC CGCGAGCACAG

**Selector probes and synthetic targets**	**Sequences (5´ - 3´)**
InfvRNAF5 biotemp	Biotin – TTTTTCAAAGGGAAATTTCAAACAGCTGCACAAAGAACAATGGTTA
KRAS MseI selector	Biotin - TTTTTTTTTTTTAGAACATGTCACACATAAGGTTAAAACAAGATTTACCTCTATTGTTGGTTTTT
KRAS synthetic MseI fragment	TAACCTTATGTGTGACATGTTCTAATATAGTCACATTTTCATTATTTTTATTATAAGGCCTGCTGAAAATGA CTGAATATAAACTTGTGGTAGTTGGAGCTGGTGGCGTAGGCAAGAGTGCCTTGACGATACAGCTAATTC AGAATCATTTTGTGGACGAATATGATCCAACAATAGAGGTAAATCTTGTTT
KRAS collector	Biotin - TCTCTCTCTCTCTCTCTTATAATAAAAATAATGAAAATGTTATCGTCAAGGCACTCTTGC
KRAS short synthetic fragment wild type	ACATTTTCATTATTTTTATTATAAGGCCTGCTGAAAATGACTGAATATAAACTTGTGGTAGTTGGAGCTGG TGGCGTAGGCAAGAGTGCCTTGACGATA
KRAS short synthetic fragment 12.1 mutant	ACATTTTCATTATTTTTATTATAAGGCCTGCTGAAAATGACTGAATATAAACTTGTGGTAGTTGGAGCTAG TGGCGTAGGCAAGAGTGCCTTGACGATA

**Detection probes/ sequencing library**	**Sequences (5´ - 3´)**
Allel2_Cy5	Cy5 - AGTCGGAAGTACTACTCTCT
DO wt KRAS cy3 1RCA	Cy3 - AGTCGATAGTCACGGCTACT
DP-1	Cy5 - AGTAGCCGTGACTATCGACT
DP-2	Cy3 - CCTCAATGCACATGTTTGGCTCC
DP-3	Alexa750 - UGCGUCUAUUUAGUGGAGCC
KRAS anchor	Alexa750 - UGUGGUAGUUGGAGCU
1st base A	Cy5 - NNNNNNNNA
1st base G	Cy3 – NNNNNNNNG
1st base T	FITC – NNNNNNNNT
1st base C	Tex red – NNNNNNNNC
1st base C blank	NNNNNNNNC
2nd base A	Cy5 - NNNNNNNAN
2nd base G	Cy3 - NNNNNNNGN
2nd base T	FITC - NNNNNNNTN
2nd base C	Tex red - NNNNNNNCN
2nd base C blank	NNNNNNNCN

Supplementary Note 1: Sequencing library preparation for mobile phone microscopy

We developed a simple 3-step library preparation scheme for targeted NGS that is optimally suited for mobile phone microscope read-out. In brief, selector probes, specific for DNA targets of interest (here described for KRAS) are immobilized on streptavidin-coated glass slides at high density through biotin-streptavidin bond. Extracted genomic DNA is enzymatically digested using restriction enzymes that excise the target region of interest creating a 50-250 bp long genomic fragment. Here, KRAS DNA fragments (192 bp) after MseI restriction digestion are selectively circularized through fragment end hybridization and ligation on the surface-attached selector probes (**Fig. 1e**). During this reaction, fragments are concentrated onto a small surface area on the glass slide by incubating the reactions in micro-well chambers attached to the slide, accumulating the molecules within our mobile phone microscope field of view (approximately 1 mm^2). The circularized fragments are then amplified on the surface through RCA and can then be directly sequenced using e.g., sequencing by ligation chemistry[1,2] (**Fig. 1e**).

RCPs may appear stretched when generated on modified glass surfaces, which complicates image analysis and base-calling (see **Fig. 2e** and **Supplementary Fig. 2**). To overcome this limitation we increased the RCP integrity through compaction oligonucleotides[3] added during RCA resulting in higher RCP integrity and improved image acquisition and analysis of sequencing reactions (**Supplementary Fig. 2**).

Supplementary Note 2:

Tumor tissues are heterogeneous and may comprise regions with KRAS mutant cells and KRAS wild type regions. Imaging a few randomly chosen ROIs may result in insufficient detection of KRAS mutant RCPs leading to a wrong genotype score. We investigated the minimal number of images of randomly chosen regions that is required to detect sufficient number of mutant RCPs in the 3 mutant samples and score these samples in concordance with clinical NGS analysis and whole tissue scanning. For this purpose, we plotted the mutant/wild type RCP ratios per ROI, sorted from low to high, and the accumulative ratio, sorted from low to high for all 3 KRAS mutant tumor samples (**Supplementary Fig. 11**). The threshold, above which a sample was scored as mutant, was set at 8 % mutant/wildtype ratio. This threshold was set above the mutant/wildtype ratio of wildtype sample E1, which showed the highest mutant/wildtype ratio of all wild type samples in whole tissue scan (7.8%) and mobile phone microscopy (7.2%) images (**Table 1** and **Supplementary Table 1**).

We found that imaging one randomly chosen area in tumor sample I detected sufficient number of mutant RCPs to score the sample as mutant. The accumulative mutant/wildtype ratio of 4 images of sample F, and 6 images of sample A, detected sufficient number of mutant RCPs to genotype the samples in concordance with clinical NGS data and whole tissue scans (**Table 1** and **Supplementary Table 1**). These results suggest that imaging a few randomly selected areas could sufficiently cover KRAS mutant cell containing areas, and expertise on or prior knowledge about tumor morphology may not be required. However, if desired, areas of interest can also be identified based on morphology using our mobile phone's bright-field microscopy mode.

Supplementary References

1 Shendure, J. *et al.* Accurate multiplex polony sequencing of an evolved bacterial genome. *Science* **309**, 1728-1732, doi:10.1126/science.1117389 (2005).

2 Drmanac, R. *et al.* Human genome sequencing using unchained base reads on self-assembling DNA nanoarrays. *Science* **327**, 78-81, doi:10.1126/science.1181498 (2010).

3 Clausson, C. M. *et al.* Compaction of rolling circle amplification products increases signal integrity and signal-to-noise ratio. *Scientific reports* **5**, 12317, doi:10.1038/srep12317 (2015).
